# Comparative Analysis of Microcystin Prevalence in Michigan Lakes by Online Concentration LC/MS/MS and ELISA

**DOI:** 10.3390/toxins11010013

**Published:** 2019-01-01

**Authors:** Johnna A. Birbeck, Judy A. Westrick, Grace M. O’Neill, Brian Spies, David C. Szlag

**Affiliations:** 1 Department of Chemistry, Wayne State University, Detroit, MI 48202, USA; goneill04@gmail.com; 2 Department of Chemistry, Oakland University, Rochester, MI 48309, USA; brspies@oakland.edu (B.S.); szlag@oakland.edu (D.C.S.)

**Keywords:** online concentration, LC/MS/MS, microcystin, Michigan lakes, Adda-ELISA, cyanotoxins

## Abstract

Fast and reliable workflows are needed to quantitate microcystins (MCs), a ubiquitous class of hepatotoxic cyanotoxins, so that the impact of human and environmental exposure is assessed quickly and minimized. Our goal was to develop a high-throughput online concentration liquid chromatography tandem mass spectrometry (LC/MS/MS) workflow to quantitate the 12 commercially available MCs and nodularin in surface and drinking waters. The method run time was 8.5 min with detection limits in the low ng/L range and minimum reporting levels between 5 and 10 ng/L. This workflow was benchmarked by determining the prevalence of MCs and comparing the Adda-ELISA quantitation to our new workflow from 122 samples representing 31 waterbodies throughout Michigan. The frequency of MC occurrence was MC-LA > LR > RR > D-Asp^3^-LR > YR > HilR > WR > D-Asp^3^-RR > HtyR > LY = LW = LF, while MC-RR had the highest concentrations. MCs were detected in 33 samples and 13 of these samples had more than 20% of their total MC concentration from MCs not present in US Environmental Protection Agency (US EPA) Method 544. Furthermore, seasonal deviations between the LC/MS/MS and Adda-ELISA data suggest Adda-ELISA cross-reacts with MC degradation products. This workflow provides less than 24-h turnaround for quantification and also identified key differences between LC/MS/MS and ELISA quantitation that should be investigated further.

## 1. Introduction

Toxic freshwater cyanobacteria blooms have an increasingly negative impact on human and environmental health and local economies [1,2,3]. Consumption of cyanotoxins via drinking water is the primary route of exposure for most people [1,4,5]. Other routes of exposure include full and partial body immersion, inhalation of spray during recreation, and consumption of blue-green algal supplements [1,6]. Microcystins (MCs) are cyclic heptapeptides that contain the following amino acids at numbered positions (1–7): (1) d-alanine, (2) variable l-amino acid, (3) d-methylaspartic acid, (4) variable l-amino acid, (5) Adda, (6) d-glutamic acid, and (7) *N*-methyldehydroalanine. The variable amino acid positions help determine the MC name. For instance, MC-LR would have l-leucine in position 2 and l-arginine in position 4. MCs are the most frequently monitored and encountered freshwater cyanotoxins in the world with over 150 different congers reported [7,8]. The US Environmental Protection Agency (US EPA) have provided guidance for drinking water treatment by publishing a total MC health advisory (HA) for drinking water. In addition, they released a draft US EPA Ambient Water Quality Criteria for recreational waters set at 4 µg/L, which is substantially lower than the WHO recreational guidance for low and moderate health effects for MCs at 10 to 20 µg/L, respectively [9,10]. US EPA has published two standard methods for the quantification of MCs: US EPA Method 544 (liquid chromatography tandem mass spectrometry (LC/MS/MS) and solid phase extraction (SPE)) and US EPA Method 546 (Adda-enzyme-linked immunosorbent assay (Adda-ELISA)) [11,12]. The US EPA Method 544 includes manual SPE and takes at least 1.5 days to perform the analysis. A comprehensive high-throughput platform and workflow is needed to provide a rapid (less than 24 h) turnaround for MC quantitation using LC/MS/MS. Furthermore, a comparison between LC/MS/MS and Adda-ELISA is needed to determine when untargeted MC analyses must be performed.

Online concentration LC/MS/MS has been accomplished for several types of naturally occurring and anthropogenic contaminants [13,14,15,16]. Online concentration LC/MS/MS workflows include particulate removal and optimization of the SPE loading column and analytical column. In 2012, Beltran et al. published an online concentration method for six MCs (MC-RR, LR, YR, LY, LF, and LW) [17]. Fayad et al. expanded and optimized the method by adding two more cyanotoxins: cylindrospermopsin and anatoxin-a [18]. This method was used to quantitate natural waters from Quebec, Canada. Munoz altered Fayads method by removing cylindrospermopsin and anatoxin-a and adding two MCs, MC-LA, and D-Asp^3^-LR [19]. The Balast et al. method consisted of five MCs, D-Asp^3^-RR, RR, D-Asp^3^-LR, LR, and YR [20]. Based on these high throughput methods, Oritz created an online concentration LC quadrupole time-of-flight (qTOF) workflow for targeted and untargeted MCs [21]. The targeted analytes were 12 MCs and anatoxin-a. Online SPE concentration provides a high throughput workflow which offers economic and quick response time advantages compared to SPE performed by manual or automated technologies. The economic advantages are reflected in decreased use of toxins, solvents, disposables, sample handling, and workforce hours. Thee quick turnaround time reduces the time water practitioners must wait before getting data needed to make public health decisions. Our goal was to create an online concentration LC/MS/MS workflow using 12 commercially available MCs and nodularin to be used for drinking water and recreational waters.

The health risk is usually based on concentration, toxicity, and duration of exposure. However, with only 12 commercially available MCs and statistically thousands of possible congeners, concentration and toxicity studies on all congeners is impractical. The 12 MCs used in this study vary by substitutions made at positions 2 and 4 and demethylation at position 3. The lethal dose (LD_50_) in intraperitoneal injection mouse studies have been reported for 10 of the 12 MCs and were summarized by Chorus and Bartram as 50 µg/Kg for MC-LR and LA, 60–100 µg/Kg for MC-YR, LY, HtyR, HilR, and >100 µg/Kg for MC-WR, D-Asp^3^-LR, D-Asp^3^-RR, and RR [1]. The toxicity of MC-LW has not been determined, while MC-LF is qualitatively known to be toxic [1]. A more comprehensive mouse toxicity evaluation supported that MC-LR is a more potent toxin than MC-YR and RR [22]. These 12 MCs have a wide range of potency reinforcing the importance of identifying and quantifying individual MCs. High-throughput monitoring and identification of the most prevalent MCs needs to be performed to focus relevant toxicology studies. The US EPA method selected six MC congeners to quantitate, but there was little explanation as to why these six were chosen. When reviewing literature in search of LC/MS/MS analyses performed with more than six MC congeners and/or nontargeted analyses throughout the US, results were limited [23,24,25,26,27,28]. Based on US studies, D-Asp^3^MC-LR, D-Asp^3^-RR, Dha^7^-RR, and HtyR are often reported at more than 20% of the total MC concentration [23,24,29,30,31]. Furthermore, Aubel and Foss reported three unknown MCs based on LC and the interpretation of their UV spectrum with estimated concentrations between 8 and 600 µg/L using the UV molar extinction coefficient for MC-LR [23]. Finally, the Green Lake bloom reported by Teta et al. showed that the most prevalent MC was a new MC, MC-MhtyR [24]. More US studies are needed to determine which MCs are most prevalent, as well as their geographic distribution. Furthermore, a universal workflow is needed to determine when an untargeted approach is needed for identification and quantitation. 

There have been numerous cyanotoxin monitoring/occurrence studies in the US and Michigan [32]. Most of these surveys have used the Adda-ELISA and provide total MC reported as MC-LR equivalent (total MC-LR eq.) [32]. Most studies only sampled once or twice per season at a single location per waterbody, providing limited spatial and temporal coverage [32]. It is therefore likely that many bloom events are missed and the cyanotoxin problem may be more widespread than reported currently. Data from the US EPA 2007 National Lake Assessment, which featured Michigan cyanotoxin reports, literature, and methods outlined by Beaver et al. were used to select sites for the 2017 season [33]. The Michigan Department of Environmental Quality (MDEQ) reviewed six studies that monitored 232 Michigan inland lakes from 2002 to 2012 [32,33,34,35,36,37,38]. MCs were present, but at relatively low concentrations. Several of these studies documented concentrations of MCs in Michigan waterbodies consistently above 1 µg/L, but there did not appear to be wide-spread dangerous levels of cyanotoxins exceeding the draft US EPA Ambient Water Quality Criteria (>4 µg/L). Some hotspots exist, such as in the Western Basin of Lake Erie and a handful of inland lakes (e.g., Lakeville Lake and Muskegon Lake) [34,39]. Additionally, the health effects of chronic low-level exposure are unknown [32]. Harmful algal blooms (HABs) are appearing more frequently, possibly due to anthropogenic changes in climate, non-point-source pollution, and invasive mussels [40]. Increased public concern about cyanotoxins has outpaced government regulation or guidance and poses challenges for local groups and agencies charged with protecting public health.

**Figure 1:**
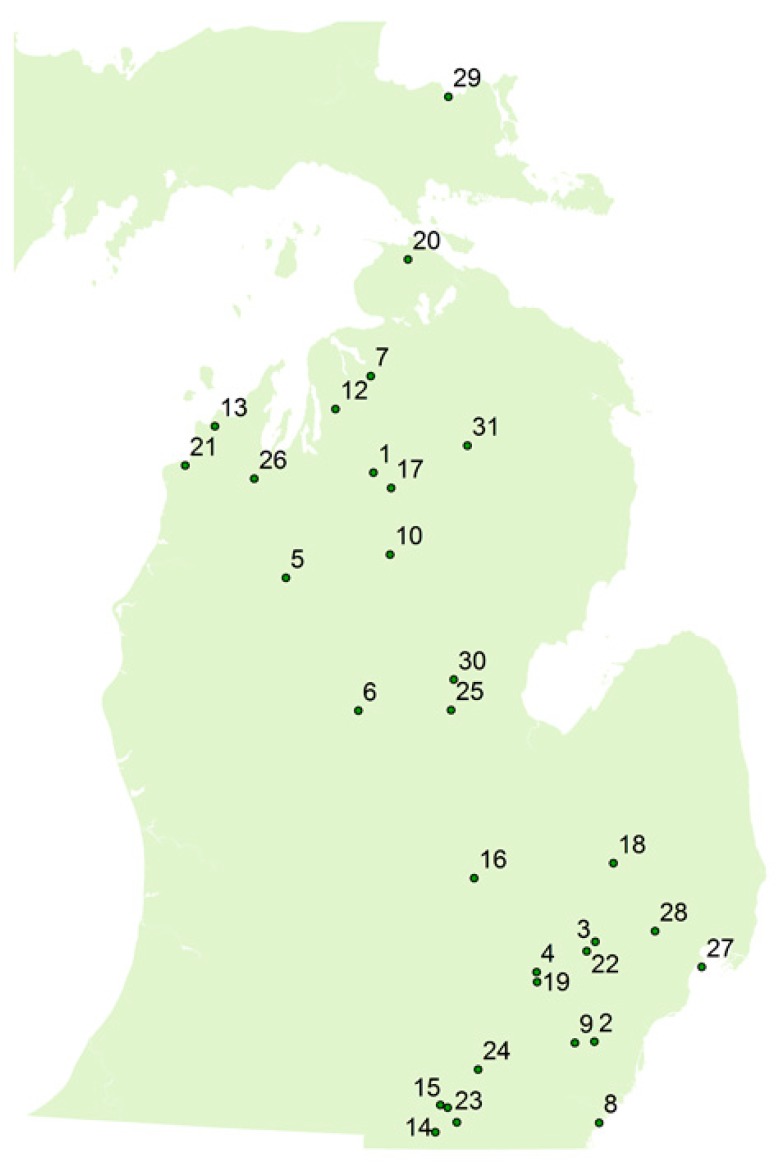
Map of Michigan and sampling sites.

Because of growing public concern about HABs, enhanced monitoring frameworks and technologies are needed to better understand the threat posed by HABs in Michigan. To this end, we sampled 31 waterbodies across Michigan in July, August, September, and October 2017 (Figure 1). Our aim was to evaluate emerging LC/MS/MS technologies against the widely used screening method based on the Adda-ELISA to identify deviations and to develop a more practical and cost effective HAB management framework. Our hypothesis was that if the LC/MS/MS method was in good agreement with the Adda-ELISA, then untargeted MC analyses would not be needed, and the distribution of targeted MCs could be used to determine the prevalent MC. The four key objectives of this study were: (1) identify the common congeners in Michigan lakes; (2) compare the total MCs determined by summing the 12 congeners measured by LC/MS/MS with the total MC-LR eq. measured by Adda-ELISA; (3) identify situations in which the LC/MS/MS and Adda-ELISA data does not agree; and (4) screen for untargeted degradation products using online concentration and full scan LC/MS.

## 2. Results and Discussion

### 2.1. Online Concentration and LC/MS/MS Methodology

In early 2015, the US EPA released the first standardized method for the detection of MC and nodularin with US EPA Method 544: Determination of Microcystins and Nodularin in Drinking Water using Solid Phase Extraction and Liquid Chromatography/Tandem Mass Spectrometry (LC/MS/MS) [11]. This method is the first to establish a standard procedure for the collection and analysis of drinking water samples for MC using LC/MS/MS. However, it does have limitations, primarily that it includes a laborious water filtration and filter extraction which makes emergency testing impossible to complete in less than 24 h. The method uses SPE cartridges to clean up and concentrate only 6 MCs (MC-RR, LR, YR, LA, LY, and LF) and nodularin in water samples. This procedure is time consuming, taking approximately an 8-h day just to prepare samples, and has the potential to introduce errors into the method process due to sample handling. Furthermore, the LC/MS/MS method has a 26 min run-time in which the first MC does not elute off until shortly after 11 min. The US EPA recognizes this; in US EPA Method 544, section 1.6 ‘Method Flexibility’, they allow users to modify LC and MS conditions due to the continuous technological advances in these techniques [11]. The sample collection and sample preparation prior to LC/MS/MS may not be modified for drinking water applications. 

Another objective of this work was to improve upon the limitations that were observed in US EPA Method 544 and demonstrate that online concentration can achieve similar results. Improvements were made by adding 6 more commercially available MCs (MC-WR, LW, HilR, HtyR, D-Asp^3^-RR, and D-Asp^3^-LR) and by utilizing the method flexibility stated in US EPA Method 544. Changes were made in both LC and MS conditions, which included mobile phase modification (0.1% formic acid in water and acetonitrile versus 20 mM ammonium formate in water and 100% methanol) and a C18 column versus a C8 column. The sample collection and processing were altered to that used in US EPA Method 546, which is suitable for ambient waters and speeds up sample processing. No preservatives were necessary because samples were frozen immediately as part of the cell lysing process.

**Figure 2:**
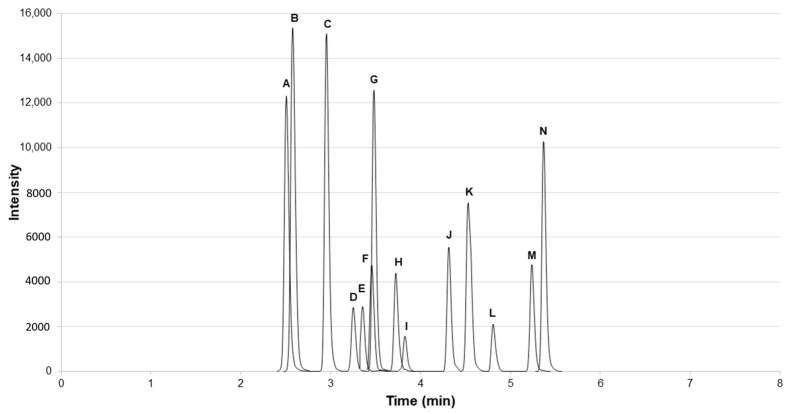
Chromatogram of selected microcystins (MCs), nodularin, and surrogate standard (L). A: D-Asp^3^-MC-RR; B: MC-RR; C: Nodularin; D: MC-YR; E: MC-HtyR; F: MC-LR; G: D-Asp^3^-MC-LR; H: MC-HilR; I: MC-WR; J: MC-LY; K: MC-LA; L: C_2_D_5_-MC-LR; M: MC-LW; and N: MC-LF.

The major innovation of this method is the elimination of the SPE by online concentration. The LC system is a two-pump system with a six-port valve to allow switching of mobile phase flows on the columns. The first configuration with the port allows mobile phases to flow to each column separately so that they can equilibrate and allows for the sample to be loaded and trapped onto the loading column and cleaned by the mobile phase (0.1% formic acid in water), which then flows into waste. One min after the injection, the valve switches positions, allowing the gradient for the analytical separation to flow through the loading column, essentially back flushing the MCs that were trapped onto the column and sending them to the analytical column for separation. The total method time is under 11 min and includes concentration, cleaning, and analytical separation. The MCs and nodularin elute off between 2–6 min, as shown in Figure 2.

### 2.2. LC/MS/MS Method Validation

To validate the online concentration LC/MS/MS method, seven fortified reagent water samples were prepared at 5 and 10 ng/L of the 12 selected MCs and nodularin. These were analyzed consecutively using the LC/MS/MS method. Using equations outlined in Table 1, the initial demonstration of precision and accuracy, method reporting limits (MRL), upper prediction interval of results (PIR) and lower PIR, and detection limits (DL) were calculated for each MC and nodularin. 

Table 2 summarizes the online concentration LC/MS/MS method validation results. The method precision and accuracy were calculated from the replicates chosen for the MRL determination. The method precision was between 2.5% and 11%, while method accuracy was between 90% and 113% for each of the selected MCs and nodularin. The higher %RSD for MC-LR may be due to the MC-LR and D-Asp^3^-LR not being completely resolved chromatographically. This method was able to achieve DLs less than US EPA Method 544 for MC-RR, YR, LR, LA, and LF, and nodularin [11]. Furthermore, this method met the criteria for setting the MRL and passed the upper and lower PIR requirements for all MCs and nodularin. The method linearity range was consistent for all MCs at 5–500 ng/L (Table 2) and expanded to a low 0.5 ng/L for select MCs and nodularin. 

### 2.3. LC/MS/MS and ELISA Method Results and Comparison

**Figure 3:**
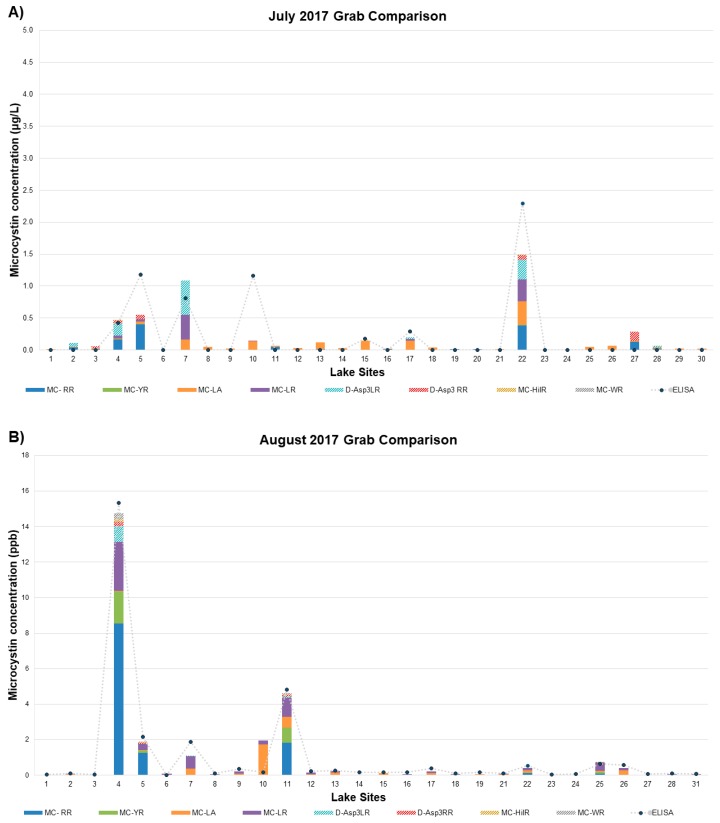

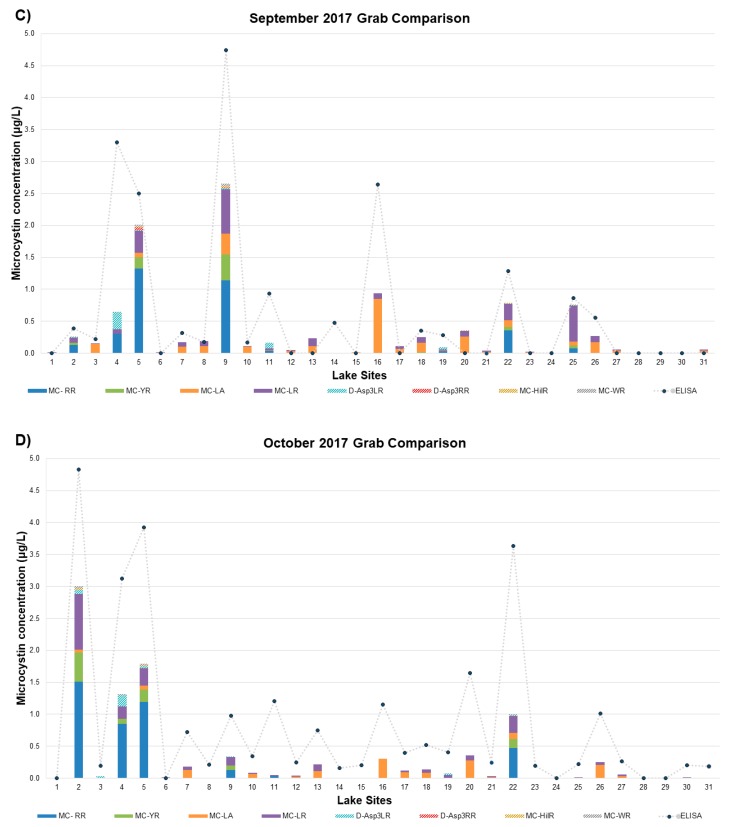
2017 sampling period; (**A**) July, (**B**) August, (**C**) September, and (**D**) October. Data comparison of total MC (µg/L) detected by LC/MS/MS (bars) and Adda-enzyme-linked immunosorbent assay (Adda-ELISA) (dots).

There have been limited studies investigating the prevalence of MC congeners in specific waterbodies in California, Illinois, Ohio, and Washington, but there has not been a thorough study of the spatial and temporal changes of MCs regionally throughout a bloom season [23,24,26,29,30,31]. The current study gives an insight into the types of MC congeners present in Michigan, as well as the changes that occur throughout a bloom season. We selected 31 waterbodies across the state of Michigan to sample monthly from July–October in 2017 (Figure 1). Paired samples were analyzed by LC/MS/MS and Adda-ELISA. The Adda-ELISA method is an indirect and competitive ELISA assay in which the Adda moiety, present in all MCs and nodularin, is detected. This assay will detect all variants of MC and nodularin and potentially degradation products containing the Adda group. Consequently, Adda-ELISA results are reported as total MC-LR eq. since the test is standardized against one congener, MC-LR. To compare the Adda-ELISA to the LC/MS/MS, we sum the measured concentrations of the 12 congeners. The monthly results are shown in Figure 3A–D, and results from the Adda-ELISA and LC/MS/MS analysis were compared for each month. As shown in Figure 3B, the August sampling period had the highest concentration of total MC detected out of the four months that this study was conducted but had the least diversity of MC congeners identified compared to the other months (Table 3). The Adda-ELISA and LC/MS/MS total MC data generally agreed for July and August and correlated well for all sampling sites. There is much greater deviation in the months of September and October although the trends are consistent.

Table 3 shows a summary of the MC congener frequency across the sample sites collected during the four sampling periods based off the MRL for each method (Adda-ELISA MRL was 150 ng/L and see Table 2 for all MC MRLs). In Table 3, the LC/MS/MS data shows that MC-LA, LR, and RR were observed most frequently out of the 12 congeners and that MC-LA and LR were observed in more samples in comparison to MC-RR. However, MC-RR was detected at higher concentrations in comparison to MC-LA and MC-LR for each month except July (see Tables S3–S6). The high concentration congeners for each month were as follows: (1) in July MC-RR, LR, D-Asp^3^-LR, and LA were each greater than 0.5 µg/L in concentration, (2) in August MC-RR, YR, LR, D-Asp^3^-LR, and LA were each 1.2 µg/L or higher in concentration, and (3) September and October MC-RR, YR, LR, LA were each above 0.5 µg/L in concentration (see Tables S5 and S6). Of these, only D-Asp^3^-MC-LR is not one of the six MC listed in US EPA Method 544 [11].

**Figure 4:**
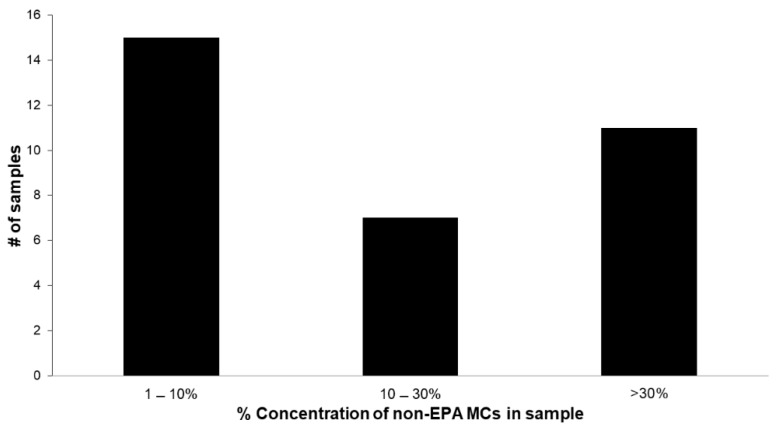
Percentage of non-US EPA Method 544 MCs detection in samples.

Figure 4 shows that over the course of the season, 33 samples had MC quantitation above the MRL, of which 15 samples contained non-EPA MC at less than 10% of total MC concentration, 7 samples contained 10% to 30% total concentration of non-EPA-MC, and 11 samples contained more than 30% total mass as non-EPA-MC. This supports the need for an expanded congener list for future US EPA methods. As stated above, the US EPA is considering a recreational ambient water quality criteria for MCs at 4 µg/L total MC. At this level, results from the Adda-ELISA would have issued four warnings during the bloom season (two in August, one in September, and one in October) and LC/MS/MS results would have issued three (all in the month of August) (Figure 3).

The Adda-ELISA and LC/MS/MS data trend similar to one another in the months of July and August (Figure 3A,B) but start to deviate in September and October (Figure 3C,D), where the Adda-ELISA data is showing higher amounts of total MCs detected in comparison to the LC/MS/MS data. The Adda-ELISA has three weaknesses: (1) the calibration is non-linear; (2) different congeners have different cross-reactivities that deviate significantly from 1.0 leading to significant over or under prediction of Total MC Equivalents; and (3) it will react with MC degradation products in some cases, leading to over prediction of MC concentrations. 

**Figure 5:**
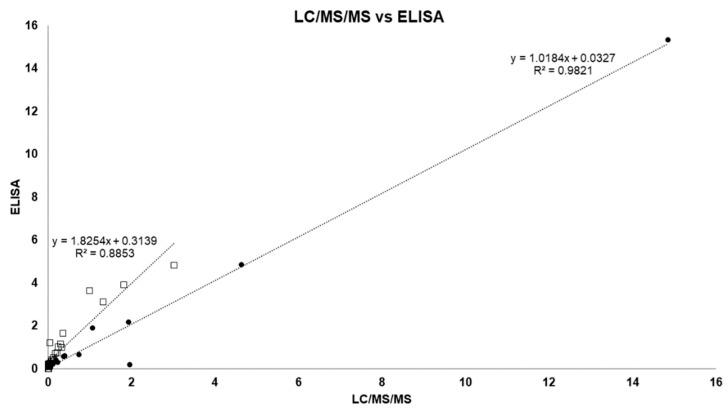
Comparison of ELISA and MS/MS for the months of August (●) and October (□).

Figure 5 shows the ratio between Adda-ELISA and LC/MS/MS for the months of August and October. In the month of August (●), the slope was approximately one with good correlation. In October the slope increased to almost 2 and the coefficient of determination decreased. We hypothesize that this is due to two factors: (1) there is the cross-reactivity of the different MCs to the Adda-ELISA and (2) there is detection of MC degradation products.

Though the Adda-ELISA is often preferred because it provides a measure of “Total” MC while LC/MS/MS can only quantify MCs with standards, it is widely known that there are varying levels of cross-reactivity between the Adda-ELISA method and the different MC congeners [41,42,43]. Depending on the MC present in the sample, the level of cross-reactivity can affect the interpretation of MC concentrations reported by either under or overestimating the total MC-LR eq. sample concentration. The most frequently occurring three MCs that were observed throughout the study were MC-LR, RR, and LA. Fischer reported that the cross-reactivity of MC-RR with the Adda-ELISA kits is 50% relative to MC-LR [42]. This would give an underestimation of total MC-LR eq., as MC-RR has the highest reported concentration as reported by LC/MS/MS. For example, in July 2017, site 27 had a total MC concentration of 0.3 µg/L by LC/MS/MS, but was not detected by Adda-ELISA. The total MC concentration consisted of MC-RR and D-Asp^3^-RR (cross-reactivity of 50% and 80%, respectively), both of which give an under-estimation of the concentration rather than an overestimation as indicated in Figure 3 and Figure 5 [42]. This suggests that the higher reported concentration in September and October may be due to the accumulation of MC biodegradation products or other interferents. 

Multiple studies have investigated the biodegradation of MCs (see reviews by Li and Dziga) [44,45]. The study by Bourne et al. identified bacteria that may be responsible for the hydrolysis of MC-LR [46]. They were able to identify two biodegradation products of MC-LR using LC/MS/MS, a linearized form of MC-LR (1013 *m*/*z*), and a tetrapeptide (615 *m*/*z*, Adda-Glu-Mdha-Ala), both of which contained the Adda group [46]. Furthermore, upon UV analysis of both compounds, Bourne observed the characteristic Adda peak at 238 nm. Removal of the Adda group or a removal of the diene double bonds would eliminate the signature 238 nm peak, suggesting the Adda group was still intact in these biodegradation products [46]. Thees et al. used LC/MS/MS to show that specific bacteria degraded MC-LR, while the Adda-ELISA showed no biodegradation [47]. Further investigation of these samples showed the presence of MC-LR degradation products, which included the tetrapeptide [47]. Additionally, Guo and colleagues have shown that the Adda-ELISA is capable of detecting MC oxidation breakdown products [41]. In their study, they showed that after oxidation of MC-LR and MC-LA with chlorine and chloramines, the Adda-ELISA measured concentrations at least two times higher than the LC/MS/MS. Further analysis of sampling sites 2, 4, 5, 16, and 22 for the month of October using full scan LC/MS showed MC degradation products 835 *m*/*z* (MC-LR double bond cleavage), 981 *m*/*z* (demethylation of MC-LR), 512 *m*/*z* (+2 demethylation of MC-RR) as well as other known MC fragments 163 and 135 *m*/*z* ([C_11_H_14_O + H]+ and [Phenol-CH_2_-CHOMe]+, respectively) [48,49,50]. High resolution MS must be completed in order to confirm the exact mass and chemical formulas of these breakdown products. With this knowledge, it is reasonable to assume that as a bloom senesces, the amount of biodegradation products increases, causing the Adda-ELISA quantitation to overestimate MCs.

## 3. Conclusions

An online concentration method for the detection of MC and nodularin was developed for fast water monitoring based off US EPA Method 544. This method has the ability to detect all MCs in the EPA method, but added an additional six MCs that are commercially available. It is able to meet or exceed the DL of the US EPA method and shows good method reproducibility and accuracy. It removes the need for SPE and drastically reduces sample handling time and analysis time, making emergency sampling feasible (completed in less than 24 h).

Throughout the state of Michigan, 31 waterbodies were sampled between the months of July and October in 2017. The most commonly found MCs were MC-LA, LR, RR, D-Asp^3^-LR, and YR, with D-Asp^3^-MC-LR being the only one not in US EPA Method 544. The Adda-ELISA results for total MCs were consistent amongst the sites for the months of July and August but started to diverge in the months of September and October, in which the Adda-ELISA results were reported to be greater than the LC/MS/MS results. This is believed to be caused by the accumulation of biodegradation products of the MCs in the lakes late in the season.

## 4. Materials and Methods

Field procedures were based on modifications of US EPA’s Survey of the Nation’s Lakes Field Operations Manual [51]. Inland lakes were selected across a south to north gradient to reflect different geologies and ecoregions. All lakes were larger than 10 ha. The sampling location on each lake was dictated by our ability to sample by wading and by our ability to gain access. The Great Lakes sites included Brimley Bay (SUP: Lake Superior), Lake St. Clair Metro Park (STC: Lake St. Clair), and Lake Erie Metro Park (ERI: Northwest Lake Erie). Water grab samples were collected by wading or from a dock at each site. Samples for toxin analysis were collected in PETG bottles to prevent adsorption and to minimize artificially ‘low’ results when toxins were present at higher concentrations. Samples were frozen within six hours.

### 4.1. Chemicals and Reagents

Water, acetonitrile, methanol, and formic acid were all Optima LC/MS grade solvents and purchased from Fisher Scientific (Tewksbury, MA, USA). MCs MC-LR, RR, YR, WR, HtyR, HilR, D-Asp^3^-RR, D-Asp^3^-LR, LA, LF, LY, LW, and Nodularin were purchased from Enzo Life Sciences, Inc. (Farmingdale, NY, USA). The surrogate C_2_D_5_ MC-LR was purchased from Cambridge Isotope Laboratories, Inc. (Tewksbury, MA, USA). The Microcystin-Adda ELISA kit was purchased from Abraxis, Inc. (Warminster, PA, USA).

Standards were prepared in methanol and diluted to ng/L concentrations in LC MS grade water. All water samples were collected according to US EPA method 546: Determination of Total Microcystins and Nodularins in Drinking Water and Ambient Water by Adda Enzyme-Linked Immunosorbent Assay. Briefly, for ambient waters, EPA Method 546 does not require any preservatives to be added to the sample collection containers [12]. A 40 mL lake water sample was collected in PETG bottle at approximately 30 cm below the surface of the water. Samples were stored on ice after collection and until arrival to the lab where they were then frozen at −8 °C. MCs were released from the cells by subjecting the samples to a series of three freeze thaw cycles and debris was removed by filtering through a 1 µm glass fiber filter [18,21,23]. 

### 4.2. ELISA

The Microcystins-Adda ELISA kit was run according to the kit directions. All Adda-ELISA kits were stored at 4 °C until used; all analyses were performed in duplicate with kits from the same lot number. Reagents were allowed to come to room temperature before beginning the assay. Briefly, 50 µL of each standard (0.15 to 5.0 µg/L) and sample was added to the ELISA wells using a multichannel pipet. Next, 50 µL of the antibody solution was added to each well. The plate was covered with parafilm and carefully swirled for 30 s and left to incubate for 90 min in the dark. After incubation, the parafilm was removed and the contents were decanted off. The plate was then washed using 250 µL of the 1× wash buffer solution three times. Next, 100 µL of the enzyme conjugate solution was added to each individual well, the plate was parafilmed and carefully swirled and allowed to incubate for another 30 min. The plate was then decanted as before and washed with the same three step procedure as above. Then, after 100 µL of the substrate color solution was added to each well, the plate parafilmed and swirled gently for another 30 s. The plate was allowed to incubate for a further 25 min in the dark before 50 µL of stop solution was added to each well. The plate was read at 450 nm using a Synergy H1 microplate reader (BioTek, Winooski, VT, USA) and data analyzed using the accompanying Gen5 2.04 software with a 4-parameter standard curve. 

### 4.3. Liquid Chromatography Mass Spectrometry

The LC/MS/MS system consisted of a Thermo Scientific TSQ Quantiva™ triple quadrupole mass spectrometer (Waltham, MA, USA) with an EQuan MAX Plus™ system. Standards and samples were loaded onto a loading column (Thermo Scientific Hypersil GOLD aQ 2.1 × 20 mm, 12 μm) using an HTC PAL autosampler (CTC Analytics, Zwingen, Switzerland) equipped with a 5 mL sampling syringe, at a flow rate of 1.5 mL/min using 0.1% formic acid in water. The system had a pre-equilibration period of 1.8 min before loading of sample onto the column. The sample was loaded onto the concentrator column and the column washed with mobile phase for the first minute. Then, after 1 min, the switching valve enabled the loading column to be back flushed with the analytical column gradient onto the analytical column (Thermo Accucore aQ, 50 × 2.1 mm, 2.6 µm), where the analytes were separated in under 6 min. For identification of unknown MCs, a full scan mode was used with a Q1 scan from 100–1300 *m*/*z*.

Two LC programs were used, one for the concentrator system and one for analytical separation. Samples were concentrated on the concentrator column with 0.1% formic acid in water at a flow rate of 1.5 mL/min for 1 min, as stated above. The analytical separation gradient consisted of 0.1% formic acid in water (mobile phase A) and 0.1% formic acid in acetonitrile (mobile phase B). At a flow rate of 0.6 mL/min, the gradient started at 24% B and was held for 0.7 min. It was then ramped to 26% B from 0.7–1.7 min, from 26–50% B from 1.7–5.5 min. The column was washed at 98% B from 5.51–6.5 min at a flow rate 1.0 mL/min, then brought back down to original conditions to equilibrate. The total run time was 8.5 min. Injection volumes were 1 mL and the column oven temperature was held at 35 °C.

MS analysis was performed using a TSQ Quantiva triple quadrupole mass spectrometer with an electrospray ionization source (ESI). The MS source settings were as follows: spray voltage was set to 3500 V, ion transfer tube temperature was set to 356 °C, vaporizer temperature at 420 °C, sheath gas pressure (arb) at 52, auxiliary gas pressure (arb) at 16, sweep gas (arb) at 2, collision gas at 1.5 mTorr, and Q1/Q3 resolution set to 0.7 Da.

For each standard, a quantitative and qualitative ion transition were chosen for use with selected reaction monitoring (SRM). Each quantifier and qualifier ion were chosen for intensity as well as being a known fragment for each specific MC [52,53]. These masses were further checked using high resolution MS. Table 4 provides a list of the SRM transitions for each standard used in the method. Quantitation was accomplished using TraceFinder™ EFS 4.1 (Version 4.1, Thermo Fisher Scientific, Waltham, MA, USA, 2016). Qual ions were visually inspected to ensure the proper MC was detected. The standard chromatogram is shown in Figure 2.

Quality control procedures were also completed when running sample sets. For every 10 samples, a positive control, negative control, fortified sample, and duplicate sample were run. Every sample and standard contained the surrogate standard to test for matrix effects and method reproducibility. The surrogate standard was added to each standard and sample. Recovery of the surrogate was tested according to US EPA Method 544 and fell within the limits of 60–130%.

## Figures and Tables

**Table 1:** Equations used for method validation.

Validation Procedure	Equation
Initial demonstration of precision: relative standard deviation (%RSD)	$\%RSD=100\times \frac{\sigma }{\left|\bar{x}\right|}$
Initial demonstration of accuracy: percent recovery (%Rec)	$\%Rec=100\times \frac{\bar{x}}{\mathrm{True}\;\mathrm{value}}$
MRL confirmation using the Half Rang for the prediction interval of results (HR_PIR_)	HR_PIR_ = 3.963*σ*
	Upper PIR limit
	$\frac{\bar{x}+HR_{PIR}}{\mathrm{Fortified}\;\mathrm{Concentration}}\times 100\leq 150\%$
	Lower PIR limit
	$\frac{\bar{x}-HR_{PIR}}{\mathrm{Fortified}\;\mathrm{Concentration}}\times 100\%\geq 50\%$
Detection Limit (DL) determination	$DL=\sigma \times t_{\left(n-1,\;1-\alpha =0.99\right)}$

*σ* = standard deviation; $\bar{x}$ = mean; *n* = number of replicates; *t*_(*n*−1, 1−*α* = 0.99)_ = Student’s *t* value for the 99% confidence level with *n* − 1 degrees of freedom.

**Table 2:** Comparison of online concentration liquid chromatography tandem mass spectrometry (LC/MS/MS) method and US EPA Method 544 validation.

Compound	DL (ng/L)	EPA 544 DL (ng/L) [11]	MRL (ng/L)	Upper PIR	Lower PIR	*r* ^2^	%RSD	%Rec
D-Asp^3^-MC-RR	0.6		5	122.46	98.32	0.9994	2.76	110
MC-RR	0.6	1.2	5	118.66	93.98	0.9998	2.93	106
Nodularin	0.9	1.8	10	104.89	85.45	0.9999	2.58	95
MC-YR	3.6	4.6	10	147.37	70.86	0.9980	8.85	109
MC-HtyR	2.6		10	117.91	62.17	0.9996	7.81	90
MC-LR	3.8	4.3	10	131.90	50.57	0.9988	11.25	91
D-Asp^3^-MC-LR	1.7		5	141.78	70.82	0.9996	8.42	106
MC-HilR	3.3		5	139.94	68.82	0.9987	8.60	104
MC-WR	2.0		5	144.79	74.78	0.9991	8.04	110
MC-LA	3.3	4.0	10	135.37	65.77	0.9981	8.73	101
MC-LY	2.6	2.2	10	139.18	84.08	0.9986	6.23	111
MC-LW	3.2		10	137.61	68.33	0.9994	8.49	102
MC-LF	1.2	3.4	5	138.41	88.53	0.9993	5.55	113

DL = detection limits; MRL = minimum reporting level; PIR = prediction interval of results; %*RSD* = Relative standard deviation; %*Rec* = Percent recovery.

**Table 3:** Summary of the number of times the 12 selected MCs were identified in 122 samples throughout the sampling period.

Date	D-Asp^3^ MC-RR	MC-RR	MC-YR	MC-HtyR	MC-LR	D-Asp^3^-MC-LR	MC-HilR	MC-WR	MC-LA	MC-LY	MC-LW	MC-LF	MS SUM	ELISA MC-LR eq.
07/17	5	14	0	0	5	6	2	0	24	0	0	0	56	7
08/17	4	11	6	2	21	5	5	4	21	1	1	1	82	16
09/17	2	8	6	1	22	7	4	2	19	0	1	0	72	17
10/17	0	7	5	1	20	7	2	4	14	1	0	0	61	27
Total	11	40	17	4	68	25	13	10	78	2	2	1	271	67

**Table 4:** Precursor, quant, and qual ions for the 12 selected MCs, nodularin, and the surrogate standards.

Compound	Precursor (*m*/*z*)	Quant ions (*m*/*z*)	Qual ions (*m*/*z*)
D-Asp^3^-MC-RR	512.861	135.071	375.054
MC-RR *	519.850	135.070	440.130
Nodularin *	825.383	135.111	389.111
MC-LA *	910.365	375.054	135.050
D-Asp^3^-MC-LR	981.430	135.111	375.111
MC-LF *	986.365	852.286	478.214
MC-LR *	995.378	135.039	213.050t
MC-LY *	1002.304	494.214	868.286
MC-HilR	1009.461	135.111	213.054
MC-LW	1025.639	517.214	891.286
C_2_D_5_ MC-LR *	1028.743	135.097	163.083
MC-YR *	1045.639	213.032	136.222
MC-HtyR	1059.426	135.111	617.222
MC-WR	1068.452	135.097	626.183

* MCs and nodularin used in US EPA method 544 [11].

## Supplementary Materials

The following are available online at http://www.mdpi.com/2072-6651/11/1/13/s1, Table S1: Lake Site Identification and Characterization, Table S2: LC/MS/MS method validation, Table S3: July 2017 LC/MS/MS data, Table S4: August 2017 LC/MS/MS data, Table S5: September 2017 LC/MS/MS data, Table S6: October 2017 LC/MS/MS data, and Table S7: Adda-ELISA data.
