# Supplementary material for: Comparative Analysis of Microcystin Prevalence in Michigan Lakes by Online Concentration LC/MS/MS and ELISA
Source: Toxins (Basel). 2019 Jan 1;11(1):13. doi: 10.3390/toxins11010013 (PMC6356304; doi:10.3390/toxins11010013)
Supplement: Supplementary file 1 [file toxins-11-00013-s001.pdf]

Supplementary Materials: Comparative Analysis of Microcystin Prevalence in Michigan Lakes by Online Concentration LC/MS/MS and ELISA

Johnna A. Birbeck [1,*], Judy A. Westrick [1,*], Grace M. O'Neill [1], Brian Spies [2] and David C. Szlag [2]

Table S1. Lake Site Identification and Characterization.

Site Number	Lake Name	Site Code	County	Hydraulics Open/Closed	Level III Ecoregion
1	Bear Lake	BEA	Kalkaska	Closed	Northern Lakes and Forests
2	Belleville Lake	BEL	Wayne	Open	Huron/Erie Lake Plains
3	Bogie Lake	BOG	Oakland	Closed	Southern Michigan/Northern Indiana Drift Plains
4	Brighton Lake	BRI	Livingston	Open	Southern Michigan/Northern Indiana Drift Plains
5	Lake Cadillac	CAD	Wexford	Open	Northern Lakes and Forests
6	Coldwater Lake	COL	Isabella	Open	Northern Lakes and Forests
7	Deer Lake	DEE	Charlevoix	Closed	North Central Hardwood Forests
8	Lake Erie	ERI	Monroe	Open	Great Lake System
9	Ford Lake	FOR	Washtenaw	Open	Huron/Erie Lake Plains
10	Houghton Lake	HOU	Roscommon	Open	Northern Lakes and Forests
11	Hudson Lake	HUD	Lenawee	Open	Eastern Corn Belt Plains
12	Intermediate lake	INT	Antrim	Open	North Central Hardwood Forests
13	Little Glen Lake	LGL	Leelanac	Open	North Central Hardwood Forests
14	Lime Lake	LIM	Hillsdale	Open	Eastern Corn Belt Plains
15	Little Round Lake	LRO	Lenawee	Closed	Eastern Corn Belt Plains
16	Manitou Lake	MAN	Shiawassee	Open	Southern Michigan/Northern Indiana Drift Plains
17	Lake Margrethe	MAR	Crawford	Open	Northern Lakes and Forests
18	Lake Nepessing	NEP	Lapeer	Closed	Southern Michigan/Northern Indiana Drift Plains
19	Ore Lake	ORE	Livingston	Open	Southern Michigan/Northern Indiana Drift Plains
20	Paradise Lake	PAR	Emmett	Open	Northern Lakes and Forests
21	Platte Lake	PLA	Benzie	Open	North Central Hardwood Forests
22	Pontiac Lake	PON	Oakland	Open	Southern Michigan/Northern Indiana Drift Plains
23	Posey Lake	POS	Lenawee	Open	Eastern Corn Belt Plains
24	Round Lake	ROU	Lenawee	Open	Southern Michigan/Northern Indiana Drift Plains

25	Sanford Lake	SAN	Midland	Open	Huron/Erie Lake Plains
26	Silver Lake	SIL	Grand Traverse	Closed	North Central Hardwood Forests
27	Lake St. Claire	STC	Macomb	Open	Great Lake System
28	Stony Creek Lake	STO	Oakland	Open	Southern Michigan/Northern Indiana Drift Plains
29	Lake Superior	SUP	Chippewa	Open	Great Lake System
30	Wixom Lake	WIX	Gladwin	Open	Northern Lakes and Forests
31	West Twin Lake	WTL	Montmorency	Open	Northern Lakes and Forests

Table S2. LC/MS/MS method validation.

Analyte	D-Asp3-RR	RR	Nodularin	YR	HtyR	LR	D-Asp3-LR	HilR	WR	LA	LY	LW	LF
1	5.448	5.453	10.016	9.633	8.558	9.101	5.627	9.522	4.749	10.322	11.539	9.215	5.939
2	5.683	5.289	9.415	10.98	9.887	10.128	4.514	11.168	5.722	11.078	11.07	9.051	5.422
3	5.695	5.081	9.257	9.574	8.81	8.206	5.318	9.655	5.418	10.998	10.84	10.618	5.416
4	5.383	5.161	9.547	11.781	9.823	10.771	5.122	11.066	5.726	9.917	10.206	10.477	5.901
5	5.394	5.314	9.445	11.769	8.564	8.335	5.24	11.509	5.831	9.75	10.535	10.496	5.243
6	5.372	5.4	9.361	10.912	9.362	9.255	5.431	10.793		9.848	11.901	11.537	5.728
7	5.662	5.515	9.578	11.731	8.025	8.068	5.953	9.353		8.485	12.052	10.682	6.066
Mean	5.52	5.32	9.52	10.91	9.00	9.12	5.32	10.44	5.49	10.06	11.16	10.30	5.67
Std Dev	0.15	0.16	0.25	0.97	0.70	1.03	0.45	0.90	0.44	0.88	0.70	0.87	0.31
HRpir	0.60	0.62	0.97	3.83	2.79	4.07	1.77	3.56	1.75	3.48	2.75	3.46	1.25
DL	0.56	0.58	0.91	3.58	2.61	3.80	1.66	3.33	2.03	3.26	2.58	3.24	1.17
Upper PIR	122.46	118.66	104.89	147.37	117.91	131.90	141.78	139.94	144.79	135.37	139.18	137.61	138.41
Lower PIR	98.32	93.98	85.45	70.86	62.17	50.57	70.82	68.82	74.78	65.77	84.08	68.33	88.53
% RSD (IDP)	2.76%	2.93%	2.58%	8.85%	7.81%	11.25%	8.42%	8.60%	8.04%	8.73%	6.23%	8.49%	5.55%
%Recovery (IDA)	110%	106%	95%	109%	90%	91%	106%	104%	110%	101%	112%	103%	113%
student *t* value	3.707	3.707	3.707	3.707	3.707	3.707	3.707	3.707	4.604	3.707	3.707	3.707	3.707
r^2	0.9994	0.9998	0.9999	0.998	0.9996	0.9988	0.9996	0.9987	0.9991	0.9981	0.9986	0.9994	0.9993
Range (ppt)	5-500	5-500	5-500	5-500	5-500	5-500	0.5-500	1-500	5-500	5-500	1-500	5-500	1-500

Table S3. July 2017 LC/MS/MS data.

July 2017													
**Sample Name**	**Site ID**	**D-Asp3-RR (ppt)**	**MC- RR (ppt)**	**MC-YR (ppt)**	**MC-HtyR (ppt)**	**MC-LR (ppt)**	**D-Asp3-LR (ppt)**	**MC-HilR (ppt)**	**MC-WR (ppt)**	**MC-LA (ppt)**	**MC-LY (ppt)**	**MC-LW (ppt)**	**MC-LF (ppt)**
BEA	1	ND	BDL	BDL	ND	ND	ND	ND	ND	15.63	ND	ND	ND
BEL	2	ND	36.49	BDL	BDL	ND	58.60	ND	ND	12.45	ND	ND	ND
BOG	3	32.36	10.96	BDL	ND	ND	BDL	ND	ND	18.26	ND	ND	ND
BRI	4	46.19	163.58	7.42	BDL	43.38	195.39	5.00	ND	7.65	ND	ND	ND
CAD	5	75.65	405.12	7.49	9.39	35.28	ND	ND	ND	28.89	ND	ND	ND
COL	6	ND	ND	ND	BDL	ND	ND	ND	ND	5.24	ND	ND	ND
DEE	7	ND	ND	ND	BDL	392.10	534.95	ND	ND	160.27	ND	ND	ND
ERI	8	ND	ND	ND	ND	ND	ND	ND	ND	47.29	ND	ND	ND
FOR	9	ND	ND	ND	BDL	ND	ND	ND	ND	24.31	ND	ND	ND
HOU	10	ND	ND	ND	ND	9.08	ND	ND	ND	137.52	ND	ND	ND
HUD	11	ND	47.49	BDL	BDL	ND	ND	ND	ND	19.21	BDL	ND	ND
INT	12	ND	ND	ND	BDL	ND	ND	ND	ND	32.05	ND	ND	ND
LGL	13	ND	17.75	BDL	BDL	BDL	ND	ND	ND	105.70	ND	ND	ND
LIM	14	ND	ND	BDL	BDL	ND	ND	ND	ND	32.59	ND	ND	ND
LRO	15	ND	ND	BDL	BDL	ND	ND	ND	ND	146.48	ND	ND	ND
MAN	16	ND	14.73	ND	ND	ND	ND	ND	ND	11.60	ND	ND	ND
MAR	17	ND	ND	ND	BDL	24.35	32.29	ND	ND	144.14	ND	ND	ND
NEP	18	ND	ND	BDL	ND	ND	ND	ND	ND	37.64	ND	ND	ND
OER	19	ND	16.50	ND	ND	ND	ND	ND	ND	ND	ND	ND	ND
PAR	20	ND	18.21	ND	BDL	ND	ND	ND	ND	ND	ND	ND	ND
PLA	21	ND	ND	BDL	ND	ND	ND	ND	ND	14.76	ND	ND	ND
PON	22	68.25	385.20	7.85	BDL	344.66	307.81	10.01	ND	365.72	ND	ND	ND
POS	23	ND	ND	BDL	ND	ND	ND	ND	ND	5.91	ND	ND	ND
ROU	24	ND	ND	BDL	ND	ND	ND	ND	ND	ND	ND	ND	ND
SAN	25	ND	ND	BDL	BDL	ND	ND	ND	ND	45.35	ND	ND	ND
SIL	26	ND	8.51	ND	ND	ND	ND	ND	ND	57.50	ND	ND	ND
STC	27	144.81	126.58	BDL	BDL	ND	ND	ND	ND	17.66	5.19	BDL	ND
STO	28	ND	24.41	BDL	ND	ND	25.41	ND	ND	15.31	ND	ND	ND
SUP	29	ND	8.19	ND	ND	ND	ND	ND	ND	11.40	ND	ND	ND
WIX	30	ND	ND	BDL	5.29	ND	ND	ND	ND	20.04	ND	ND	ND
WTL	31	ND	ND	ND	ND	ND	ND	ND	ND	ND	ND	ND	ND

* BDL = Below Detection Limit; * ND = Not Detected.

Table S4. August 2017 LC/MS/MS data.

		August 2017											
**Sample Name**	**Site ID**	**D-Asp3- RR (ppt)**	**MC- RR (ppt)**	**MC-YR (ppt)**	**MC-HtyR (ppt)**	**MC-LR (ppt)**	**D-Asp3-LR (ppt)**	**MC-HilR (ppt)**	**MC-WR (ppt)**	**MC-LA (ppt)**	**MC-LY (ppt)**	**MC-LW (ppt)**	**MC-LF (ppt)**
BEA	1	ND	ND	ND	ND	7.61	ND	ND	ND	ND	ND	ND	ND
BEL	2	ND	30.75	ND	ND	28.77	ND	ND	ND	11.78	ND	ND	ND
BOG	3	ND	ND	ND	ND	11.03	ND	ND	ND	ND	ND	ND	ND
BRI	4	255.12	8551.74	1798.53	106.68	2763.96	902.25	149.96	302.21	11.13	ND	10.10	5.24
CAD	5	62.92	1244.86	133.92	61.46	337.41	ND	29.69	26.50	49.06	ND	ND	ND
COL	6	ND	ND	ND	ND	54.65	ND	ND	ND	21.53	ND	ND	ND
DEE	7	ND	4.82	ND	ND	679.49	29.58	ND	ND	369.87	ND	ND	ND
ERI	8	ND	6.96	ND	ND	15.82	ND	ND	ND	ND	ND	ND	ND
FOR	9	ND	21.88	ND	ND	129.05	ND	ND	ND	58.87	ND	ND	ND
HOU	10	ND	13.05	ND	ND	224.91	ND	ND	ND	1728.62	6.26	ND	ND
HUD	11	80.02	1830.26	860.62	ND	1080.87	70.89	44.57	62.45	582.95	23.59	ND	12.34
INT	12	ND	ND	ND	ND	82.93	ND	ND	ND	55.32	ND	ND	ND
LGL	13	ND	ND	ND	ND	106.00	ND	ND	ND	144.81	ND	ND	ND
LIM	14	ND	ND	ND	ND	ND	ND	ND	ND	ND	ND	ND	ND
LRO	15	ND	ND	ND	ND	ND	ND	ND	ND	127.33	ND	ND	ND
MAN	16	ND	ND	ND	ND	21.00	ND	ND	ND	29.29	ND	ND	ND
MAR	17	ND	ND	ND	ND	97.39	ND	ND	ND	102.54	ND	ND	ND
NEP	18	ND	ND	ND	ND	9.50	ND	ND	ND	21.92	ND	ND	ND
ORE	19	ND	5.99	ND	ND	ND	ND	ND	ND	4.74	ND	ND	ND
PLA	21	ND	ND	ND	ND	23.29	ND	ND	ND	39.49	ND	ND	ND
PON	22	ND	126.34	29.58	ND	116.14	ND	ND	ND	121.04	ND	ND	ND
POS	23	ND	ND	ND	ND	ND	ND	ND	ND	ND	ND	ND	ND
ROU	24	ND	ND	ND	ND	ND	ND	ND	ND	ND	ND	ND	ND
SAN	25	ND	71.97	96.93	ND	474.18	22.61	21.42	ND	62.83	ND	ND	ND
SIL	26	ND	ND	ND	ND	135.22	ND	ND	ND	277.14	ND	ND	ND
STC	27	ND	ND	ND	ND	ND	ND	ND	ND	ND	ND	ND	ND
STO	28	ND	ND	ND	ND	12.36	ND	ND	ND	11.91	ND	ND	ND
WIX	30	35.53	449.39	620.93	ND	3569.73	102.48	112.62	26.26	79.92	ND	ND	ND
WTL	31	ND	ND	ND	ND	12.31	ND	ND	ND	11.78	ND	ND	ND

* BDL = Below Detection Limit; * ND = Not Detected.

Table S5. September 2017 LC/MS/MS data.

		September 2017											
**Sample Name**	**Site ID**	**D-Asp3- RR (ppt)**	**MC- RR (ppt)**	**MC-YR (ppt)**	**MC-HtyR (ppt)**	**MC-LR (ppt)**	**D-Asp3-LR (ppt)**	**MC-HilR (ppt)**	**MC-WR (ppt)**	**MC-LA (ppt)**	**MC-LY (ppt)**	**MC-LW (ppt)**	**MC-LF (ppt)**
BEA	1	ND	ND	ND	ND	5.32	ND	ND	ND	7.31	ND	ND	ND
BEL	2	ND	127.04	36.24	ND	79.41	5.65	BDL	4.24	BDL	ND	ND	ND
BOG	3	ND	ND	ND	ND	8.42	BDL	ND	ND	149.88	ND	ND	ND
BRI	4	ND	306.23	ND	ND	72.61	263.38	ND	4.75	ND	ND	ND	ND
CAD	5	45.40	1323.79	179.07	29.86	348.09	5.50	17.02	29.49	67.22	ND	ND	ND
COL	6	ND	ND	ND	ND	10.67	ND	ND	ND	5.32	ND	ND	ND
DEE	7	ND	ND	ND	ND	69.41	ND	ND	ND	106.66	ND	ND	ND
ERI	8	ND	ND	ND	ND	81.67	ND	ND	ND	109.84	ND	42.55	ND
FOR	9	14.85	1136.22	406.86	ND	695.23	15.93	18.87	35.19	326.11	BDL	ND	ND
HOU	10	ND	ND	ND	ND	12.34	ND	ND	ND	98.77	ND	ND	ND
HUD	11	ND	40.32	ND	ND	26.19	89.57	ND	ND	9.61	ND	ND	ND
INT	12	ND	ND	ND	ND	18.38	ND	ND	ND	33.93	ND	ND	ND
LGL	13	ND	ND	ND	ND	124.70	BDL	ND	ND	111.88	ND	ND	ND
LIM	14	ND	ND	ND	ND	ND	ND	ND	ND	ND	ND	ND	ND
LRO	15	ND	ND	ND	ND	ND	ND	ND	ND	18.38	ND	ND	ND
MAN	16	ND	ND	ND	ND	87.22	ND	ND	ND	851.64	ND	ND	ND
MAR	17	ND	ND	ND	ND	42.40	ND	ND	ND	68.49	ND	ND	ND
NEP	18	ND	3.05	13.48	ND	85.27	BDL	ND	ND	148.78	ND	ND	ND
ORE	19	ND	32.89	ND	ND	22.89	34.43	ND	ND	6.18	ND	ND	ND
PAR	20	ND	ND	ND	ND	88.93	ND	4.34	ND	263.36	ND	ND	ND
PLA	21	ND	ND	ND	ND	19.70	ND	ND	ND	22.00	ND	ND	ND
PON	22	ND	353.91	56.71	4.37	255.87	ND	6.41	4.59	106.74	ND	ND	ND
POS	23	ND	ND	3.49	ND	9.69	ND	ND	ND	7.94	ND	ND	ND
ROU	24	ND	ND	ND	ND	ND	ND	ND	ND	ND	ND	ND	ND
SAN	25	ND	71.57	49.74	ND	561.48	6.40	12.91	ND	61.79	ND	ND	ND
SIL	26	ND	ND	ND	ND	101.64	ND	ND	ND	168.65	ND	ND	ND
STC	27	ND	ND	ND	ND	15.86	ND	ND	ND	38.90	ND	ND	ND
STO	28	ND	ND	ND	ND	BDL	ND	ND	ND	ND	ND	ND	ND
SUP	29	ND	ND	ND	ND	BDL	ND	ND	ND	BDL	ND	ND	ND
WIX	30	ND	ND	ND	ND	9.16	ND	ND	ND	ND	ND	ND	ND
WTL	31	ND	ND	ND	ND	15.12	ND	ND	ND	42.98	ND	ND	ND

* BDL = Below Detection Limit; * ND = Not Detected.

Table S6. October 2017 LC/MS/MS data.

		October 2017											
**Sample Name**	**Site ID**	**D-Asp3- RR (ppt)**	**MC- RR (ppt)**	**MC-YR (ppt)**	**MC-HtyR (ppt)**	**MC-LR (ppt)**	**D-Asp3-LR (ppt)**	**MC-HilR (ppt)**	**MC-WR (ppt)**	**MC-LA (ppt)**	**MC-LY (ppt)**	**MC-LW (ppt)**	**MC-LF (ppt)**
BEA	1	ND	ND	ND	ND	2.45	ND	ND	ND	ND	ND	ND	ND
BEL	2	ND	1506.29	457.57	ND	869.37	57.91	31.74	32.00	51.19	10.75	ND	ND
BOG	3	ND	ND	ND	ND	ND	20.14	ND	ND	9.73	ND	ND	ND
BRI	4	ND	847.98	84.85	ND	186.22	181.97	BDL	13.15	ND	ND	ND	ND
CAD	5	ND	1193.36	194.64	22.22	276.62	21.78	22.60	21.81	58.58	ND	ND	ND
COL	6	ND	ND	ND	ND	14.62	ND	ND	ND	ND	ND	ND	ND
DEE	7	ND	ND	ND	ND	47.51	ND	ND	ND	129.90	ND	ND	ND
ERI	8	ND	ND	ND	ND	ND	ND	ND	ND	ND	ND	ND	ND
FOR	9	ND	128.78	66.21	ND	132.95	9.80	ND	ND	ND	ND	ND	ND
HOU	10	ND	ND	ND	ND	16.23	ND	ND	ND	66.97	ND	ND	ND
HUD	11	ND	33.38	ND	ND	13.60	ND	ND	ND	ND	ND	ND	ND
INT	12	ND	ND	ND	ND	22.00	ND	ND	ND	23.02	ND	ND	ND
LGL	13	ND	ND	ND	ND	103.34	ND	ND	ND	114.63	ND	ND	ND
LIM	14	ND	ND	ND	ND	ND	ND	ND	ND	ND	ND	ND	ND
LRO	15	ND	ND	ND	ND	ND	ND	ND	ND	8.84	ND	ND	ND
MAN	16	ND	ND	ND	ND	ND	ND	ND	ND	307.52	ND	ND	ND
MAR	17	ND	ND	ND	ND	25.07	ND	ND	ND	91.59	ND	ND	ND
NEP	18	ND	ND	ND	ND	45.97	ND	ND	ND	88.89	ND	ND	ND
ORE	19	ND	5.95	ND	ND	39.49	27.04	ND	ND	ND	ND	ND	ND
PAR	20	ND	ND	ND	ND	74.22	ND	ND	ND	279.78	ND	ND	ND
PLA	21	ND	ND	ND	ND	19.33	ND	ND	ND	12.56	ND	ND	ND
PON	22	ND	474.05	134.73	ND	257.35	15.20	BDL	11.89	103.99	ND	ND	ND
POS	23	ND	ND	ND	ND	ND	ND	ND	ND	ND	ND	ND	ND
ROU	24	ND	ND	ND	ND	ND	ND	ND	ND	ND	ND	ND	ND
SAN	25	ND	ND	ND	ND	13.36	ND	ND	ND	ND	ND	ND	ND
SIL	26	ND	ND	ND	ND	36.47	ND	ND	ND	211.62	ND	ND	ND
STC	27	ND	ND	ND	ND	25.68	ND	ND	ND	29.25	ND	ND	ND
STO	28	ND	ND	ND	ND	ND	ND	ND	ND	ND	ND	ND	ND
SUP	29	ND	ND	ND	ND	ND	ND	ND	ND	ND	ND	ND	ND
WIX	30	ND	ND	ND	ND	13.29	ND	ND	ND	ND	ND	ND	ND
WTL	31	ND	ND	ND	ND	8.67	ND	ND	ND	ND	ND	ND	ND

* BDL = Below Detection Limit; * ND = Not Detected.

Table S7. Adda-ELISA data.

July			August			September			October		
**Sample Name**	**Site ID**	**Total MC-LR Eq.(ppb)**	**Sample Name**	**Site ID**	**Total MC-LR Eq.(ppb)**	**Sample Name**	**Site ID**	**Total MC-LR Eq.(ppb)**	**Sample Name**	**Site ID**	**Total MC-LR Eq.(ppb)**
BEA	1	0.05	BEA	1	0.04	BEA	1	BD	BEA	1	BD
BEL	2	0.10	BEL	2	0.11	BEL	2	0.39	BEL	2	4.83
BOG	3	0.11	BOG	3	0.04	BOG	3	0.22	BOG	3	0.20
BRI	4	0.42	BRI	4	15.32	BRI	4	3.30	BRI	4	3.13
CAD	5	1.18	CAD	5	2.15	CAD	5	2.50	CAD	5	3.92
COL	6	0.02	COL	6	0.02	COL	6	BD	COL	6	BD
DEE	7	0.81	DEE	7	1.88	DEE	7	0.32	DEE	7	0.72
ERI	8	0.01	ERI	8	0.09	ERI	8	0.17	ERI	8	0.22
FOR	9	0.14	FOR	9	0.34	FOR	9	4.75	FOR	9	0.98
HOU	10	1.17	HOU	10	0.16	HOU	10	0.17	HOU	10	0.35
HUD	11	0.12	HUD	11	4.83	HUD	11	0.93	HUD	11	1.21
INT	12	0.11	INT	12	0.23	INT	12	BD	INT	12	0.25
LGL	13	0.06	LGL	13	0.26	LGL	13	BD	LGL	13	0.75
LIM	14	0.05	LIM	14	0.15	LIM	14	0.47	LIM	14	0.16
LRO	15	0.18	LRO	15	0.18	LRO	15	BD	LRO	15	0.21
MAN	16	0.12	MAN	16	0.16	MAN	16	2.64	MAN	16	1.16
MAR	17	0.29	MAR	17	0.39	MAR	17	BD	MAR	17	0.40
NEP	18	0.08	NEP	18	0.10	NEP	18	0.36	NEP	18	0.52
ORE	19	0.09	ORE	19	0.17	ORE	19	0.28	ORE	19	0.41
PAR	20	0.11	PAR	20	>5	PAR	20	BD	PAR	20	1.65
PLA	21	0.05	PLA	21	0.10	PLA	21	BD	PLA	21	0.24
PON	22	2.30	PON	22	0.55	PON	22	1.28	PON	22	3.63
POS	23	0.10	POS	23	0.03	POS	23	BD	POS	23	0.20
ROU	24	0.10	ROU	24	0.07	ROU	24	BD	ROU	24	BD
SAN	25	0.07	SAN	25	0.63	SAN	25	0.86	SAN	25	0.22
SIL	26	0.11	SIL	26	0.58	SIL	26	0.55	SIL	26	1.01
STC	27	0.05	STC	27	0.06	STC	27	BD	STC	27	0.27
STO	28	0.09	STO	28	0.10	STO	28	BD	STO	28	BD
SUP	29	0.09	SUP	29	X	SUP	29	BD	SUP	29	BD
WIX	30	0.05	WIX	30	>5	WIX	30	BD	WIX	30	0.21
WTL	31	0.05	WTL	31	0.07	WTL	31	BD	WTL	31	0.19
